# Risk of Attacks by Blackflies (Diptera: Simuliidae) and Occurrence of Severe Skin Symptoms in Bitten Patients along the Eastern Border of the European Union

**DOI:** 10.3390/ijerph19137610

**Published:** 2022-06-22

**Authors:** Monika Sitarz, Alicja M. Buczek, Weronika Buczek, Alicja Buczek, Katarzyna Bartosik

**Affiliations:** 1 Chair and Department of Conservative Dentistry with Endodontics, Faculty of Medical Dentistry, Medical University of Lublin, 20-059 Lublin, Poland; monika.sitarz@umlub.pl; 2 Department of Biology and Parasitology, Faculty of Health Sciences, Medical University of Lublin, 20-080 Lublin, Poland; abuczek21@gmail.com (A.M.B.); wera1301@gmail.com (W.B.); katarzyna.bartosik@umlub.pl (K.B.)

**Keywords:** *Simulium*, blackfly, blackfly bites, dermatitis, simuliosis, enhanced simuliosis symptoms

## Abstract

The components of blackfly (Diptera: Simuliidae) saliva secreted during feeding on humans and animals induce various pathological reactions manifested by skin lesions and systemic symptoms. In this study, we describe 43 cases of severe skin lesions induced by blackfly bites and analyze their potential causes. Based on the results of a survey of 418 patients, we identified periods with risk of blackfly attacks and their environmental determinants in the eastern part of the European Union. Especially strong inflammatory reactions after blackfly bites were reported in patients with concomitant cardiovascular diseases (mainly with venous insufficiency), metabolic diseases, and bacterial infections. Enhanced symptoms of simuliosis were also observed in other patients attacked by these insects only once or repeatedly. The greatest number of blackfly attacks in the study area is recorded from May to July, with a peak in June (38.73%) in the afternoon (37.10%) and evening (33.03%), when patients are in fields, forests, or their households. The case analysis indicates interactions of various factors in the development of severe inflammatory reactions in patients bitten by blackflies. Therefore, it is recommended that subjects exposed to the presence of blackflies during their work or rest should limit the length of their stay in a vulnerable environment during the highest seasonal and daily activity of these insects. It is also necessary to take measures to reduce the number of blackflies and popularize methods for prophylaxis of their attacks.

## 1. Introduction

Bloodsucking arthropods are one of the most important biological factors in hypersensitivity in humans and animals [1]. They are represented by blackflies (Diptera: Simuliidae), which are characterized by a wide geographic distribution and an ability to exert a strong impact on the host.

Blackflies colonize running water ecosystems in various continents, except Antarctica [2]. They also occur abundantly in some areas of Europe from the Baltic States [3,4,5], Great Britain, and Ireland [6,7] in the north through the countries of the western [8] and central [9,10,11,12,13,14] parts of the continent to Spain [15,16] and Italy [17,18] in the south.

During the peak of their seasonal activity, adult stages may appear in massive numbers. Only blackfly females can parasitize humans and animals present outdoors via massive attacks [19]. Compounds with various chemical structures (i.e., carboxylic acids, alcohols, aldehydes, alkanes, and ketones) excreted by mammalian hosts are attractants for host-seeking female blackflies [20,21,22].

Blackflies play an important role in the development and spread of parasites, with great epidemiological importance. They are vectors of filarial nematodes from the genus *Onchocerca*, mainly *Onchocerca volvulus*, which is the etiological agent of numerous cases of human onchocerciasis (river blindness) in sub-Saharan Africa [23]. In recent years, zoonotic onchocerciasis in humans caused by parasitic *Onchocerca* species spread by blackflies has been increasingly reported from various parts of the world including Europe and Asia (e.g., [24,25,26]). So far, five species of zoonotic *Onchocerca* spp. have been identified in humans, namely *O. lupi*, *O. gutturosa*, *O. cervicalis*, *O. dewittei japonica*, and *O. jakutensis*. These commonly reside in the subcutaneous tissue and the eyeball, causing inflammation. In case of ocular invasion, patients have reported impaired vision and a moving floater, and pain. Moreover, Simuliidae are intermediate hosts for other filariae, including *Mansonella ozzardi*, which cause human mansonelliasis in Central and South America [27,28].

In their entire occurrence area, anthropophilic blackfly species pose medical problems related to the direct consequences of their parasitism, i.e., skin lesions and systemic reactions. Massive blackfly attacks, especially in agriculture and tourism regions, may lead to large economic losses associated with limitation of outdoor activities for residents and tourists [29,30,31].

For these reasons, to protect humans against blackflies it is particularly important to determine periods related to the risk of their attacks in various environments. In clinical practice, it is essential to identify factors that may influence the clinical picture of *Simulium* (blackfly) dermatitis in patients and their treatment.

Therefore, this study was carried out to examine the environmental determinants of simuliosis and the diverse course of the disease in patients. Based on cases, we analyzed the potential causes of severe symptoms induced by blackfly bites in humans. We also summarized the state of knowledge of skin lesions caused by blackfly bites.

## 2. Material and Methods

### 2.1. Ethics Statement

Ethical review and approval were waived for this study. The photographs presented in our manuscript are those of undistinctive parts of the body, and according to generally accepted ethical standards may be used without consent as long as they are anonymized and are not accompanied by text that may reveal the patient’s identity. Patients did not participate in clinical trials that would require the consent of the Bioethics Committee. This retrospective study looks backward and examines exposures to suspected risk or protection factors. As already mentioned, studies that do not affect treatment in any way do not require the consent of the Bioethics Committee and are in line with Polish law and the principles of Good Clinical Practice. All patients reported to their general practitioner after being bitten by Simuliidae to treat lesions caused by arthropod bites. Our article does not contain personal medical information about an identifiable living individual, although patients were asked to sign our consent form. Informed consent was obtained from all subjects involved in the study. The data presented in this study are available on request from the corresponding author. The data are not publicly available due to patient privacy.

### 2.2. Study Area

The study was conducted in Lubelskie Province (southeastern Poland), which is the easternmost region of the European Union. This area is located in the temperate transitional climate zone.

This region of Poland has three large rivers (Bug, Wieprz, and San) with numerous fast flowing tributaries characteristic of upland and mountain areas. There are numerous small lakes in the northern part of Lubelskie Province. Approximately 70% of the area is covered by forests separated by arable fields and meadows. The forest structure is dominated by pines. Depending on the habitat, there are also numerous fir, Carpathian beech, alder, hornbeam, or riparian alder forests. The area has many swamps and peat bogs. The topography and numerous water reservoirs offer favorable conditions for the development of arthropods that prefer high humidity.

### 2.3. Characteristics of Patients

Forty-three patients (32 females and 11 males) with severe skin signs and systemic symptoms were selected from a group of patients reporting a blackfly bite incident in outpatient clinics of southeastern Poland. This group was excluded from the other studies conducted in the same period and described in our earlier report [32].

The patients underwent a general medical examination. Their medical history included information about other co-morbidities and medications administered during treatment. The patients received medical care until resolution of the skin signs and systemic symptoms induced by blackfly bites. During subsequent visits to the outpatient clinics, the extent and severity of the skin lesions and the course of treatment were assessed.

The skin lesions and other symptoms of blackfly bites were monitored during follow-up visits, which allowed determination of their persistence in individual patients and the effectiveness of the treatment. Due to the short feeding time, it was impossible to collect blackflies from the patients’ bodies. To identify the etiological factor of the local and systemic reactions in the patients, the insects were captured using an entomological mesh in areas indicated by the patients during the medical interview. In the laboratory, the collected specimens were viewed using a stereoscopic microscope. The entomological study based on the paper by Niesiołowski and Bokłak [33] confirmed the presence of *Simulium* sp. in the areas where the patients had stayed. During the field studies, we did not collect other arthropods that could potentially attack patients in the habitat.

The data provided by the Institute of Meteorology and Water Management were used in the analysis of the correlations between the number of blackfly bites in the individual months and meteorological parameters.

### 2.4. Survey

The questionnaires were disseminated among 418 patients who visited the outpatient clinic after blackfly bite incidents during the study in 2003–2005. The questions were answered by parents in the case of underage patients. The survey consisted of two parts. The answers to the questions in the first part were presented in our earlier study [32]. In turn, the data on the circumstances of blackfly attacks on humans and the activity of the insects in the environment were used in this study. Thus, the time of the day and months of the year with the greatest risk of blackfly attacks in eastern Poland were determined. The questionnaires comprised 10 questions arranged in three subgroups. The first subgroup included questions seeking general information about the patients, e.g., their sex, age, education, and type of occupation. The second subgroup consisted of questions about the arthropod attacks, i.e., the date, circumstances (time of day, recreational/occupational activity), the area of blackfly attack (e.g., field/garden, forest, meadow, household), and characteristics of the natural living environment of the patients (presence of forest areas, rivers, and other water reservoirs).

The third subgroup contained questions about the persistence of local skin lesions in the respondents after the blackfly attacks.

### 2.5. Statistical Analysis

The statistical analysis of the persistence of skin lesions is presented as the arithmetic mean ± standard deviation, median, and the lowest and highest values of the statistical series.

The relationships between the number of bites in a given month and the monthly mean temperature, between the number of bites in a given month and the mean monthly precipitation sum, and between the number of bites in a given month and the ratio of the mean monthly temperature and the mean monthly precipitation sum were analyzed using Spearman’s rank correlation coefficient. The results were considered statistically significant at the level of *p* ≤ 0.05. They were analyzed in Statistica 6.0. Statistic for Windows. Statsoft, Tulsa, OK, USA, 2001; https://software.dell.com/register/72480 (accessed on 20 March 2006).

## 3. Results

### 3.1. Analysis of Cases of Severe Symptoms Caused by Blackfly Bites

**Figure 1:**
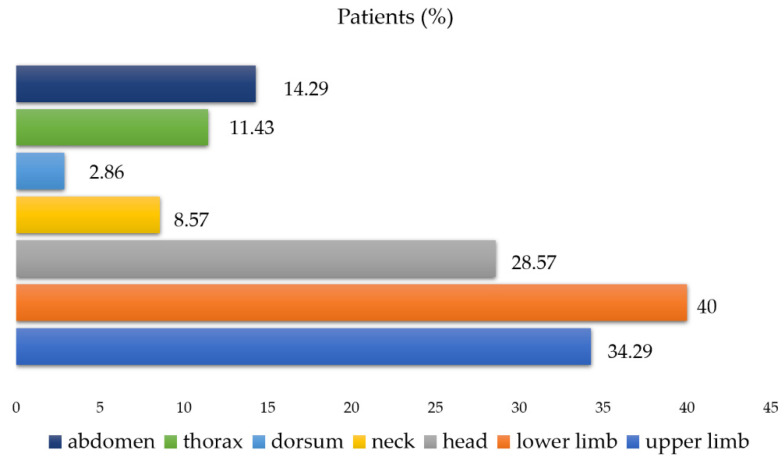
Percentage of patients related to bite location in cases of severe local reactions (*n* = 49).

**Figure 2:**
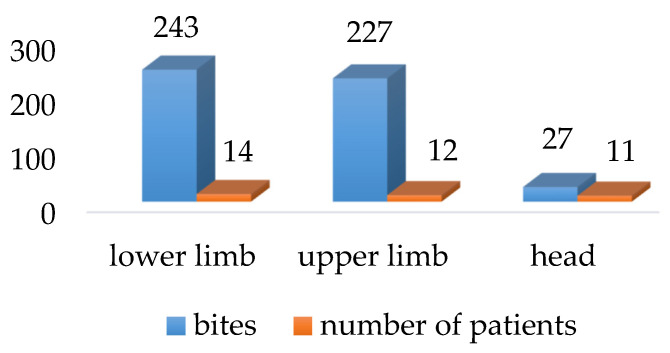
Location of black fly bites on exposed parts of the body in patients with severe local reactions (*n* = 37).

Patients with severe skin lesions were attacked simultaneously on various parts of the body, but most often on the lower (40%) and upper (34.29%) extremities and the head (28.57%) (Figure 1). The most numerous blackfly bites were observed on the lower and upper extremities (as many as 243 bites on the lower limb in 14 patients and 227 bites on the upper limb in 12 patients); a lower number of bites were visible on the head (Figure 2). The shin, forearm, forehead, and cheek were the most common exposed areas attacked by blackflies.

The age of patients with severe symptoms ranged from 4 to 65 years. The strongest inflammatory reactions were noted in patients with comorbid long-term cardiovascular diseases and metabolic disorders (cases 1 and 2).

A majority of the patients did not report any other diseases. This group included patients that had been bitten by blackflies once (cases 3 and 4) or repeatedly (cases 5 and 6). Bacterial infection was diagnosed in two patients with *Simulium* dermatitis. In one of them, the skin lesions persisted for 21 days despite the implementation of antibiotic therapy (case 7).

We selected seven of the 43 cases with severe symptoms of blackfly bites. Due to their clinical picture and the course of the disease, they seemed interesting for the obtaining of a better picture of the effects of the parasitism of these insects. We were inspired to present detailed descriptions of these cases by doctors who still have difficulties with the diagnosis and treatment of lesions caused by arthropods, especially by blackflies.

**Figure 3:**
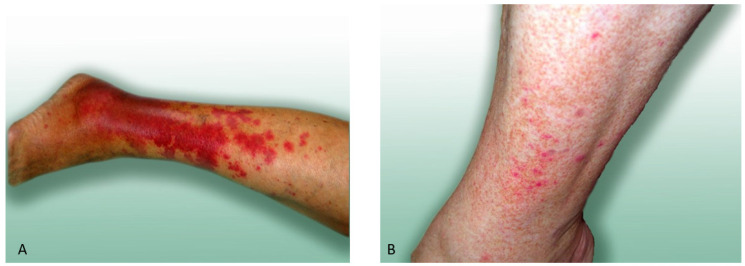
Skin lesions after blackfly bites in patients with co-morbidities. (**A**) Diffuse inflamed erythema on the leg of a patient repeatedly attacked by blackflies (case 1). The purplish color suggests vasculitic skin lesions; (**B**) Papules and vesicles on the shin of a female patient after a single blackfly bite (case 2).

**Case 1**. Female aged 65 (Figure 3A) with obesity had previously been repeatedly attacked by blackflies. She had been treated for venous insufficiency of the lower extremities for 20 years and for cardiac insufficiency and hypertension for 15 years. The patient had been exposed to chronic stress related to a very difficult family situation.

She visited a medical clinic after being bitten by blackflies in her garden located in an urbanized area. The patient was attacked by the insects in the afternoon (after 4:00 p.m.) on 25 May 2004. She was subjected to general medical examination in the clinic, which revealed numerous blackfly bites.

On the day of the blackfly attack, the patient developed extensive erythematous lesions on the shins persisting for four weeks despite symptomatic treatment. The skin lesions were accompanied by pruritus, burning sensation, hot skin on the lower extremities (both shins), pain, and tingling of the shins, which was intensified on the anterior and medial surfaces of the left leg and on the posterior and lateral surfaces and above the ankle on the right leg.

The temperature of the patient’s body was elevated for some time. On the day of the bite, the temperature was 37.8 °C. During the first week after the blackfly attack, the temperature ranged between 37.5 °C and 37.0 °C. The patient reported difficulties in mobility due to exacerbation of venous insufficiency and symptoms related to the blackfly attack.

The patient was prescribed gluco-corticosteroids: Hydrocortisone 0.1% in a single intravenous injection and Laticort 0.1% cream (containing hydrocortisone butyrate as the active substance) applied topically on the skin twice a day for four weeks as well as the antihistamine drug Zyrtec (cetirizine dihydrochloride)—1 tablet 1 × a day and Calcium—1 tablet 3 × a day.

During the incident described in this study, the patient was attacked by blackflies several times, which led to cutaneous hyperesthesia in the shins and enhanced contact-allergy reactions (erythema, itching, burning) induced by contact with grasses and other plants in the garden or park. These erythematous lesions appeared within several minutes and were less severe than those after the blackfly attack. They resolved within a few hours after topical and systemic application of the drugs. The treatment was similar to that of the blackfly bites with no intravenous infusion of Hydrocortisone 0.1%.

**Case 2**. Female aged 48 years (Figure 3B) with obesity. She had been treated for 15 years for varicose disease of the lower extremities. The patient was first attacked by blackflies while working in her garden in the afternoon on 7 July 2005.

The patient had sore and itchy papules and vesicles on the shin. The lesions persisted for 3 days.

Treatment: gluco-corticosteroid (Elocom ointment 0.1% 1 × a day), antihistamine drug Cetirizine (Zyrtec) 1 tablet a day, and Calcium 3 × a day.

**Figure 4:**
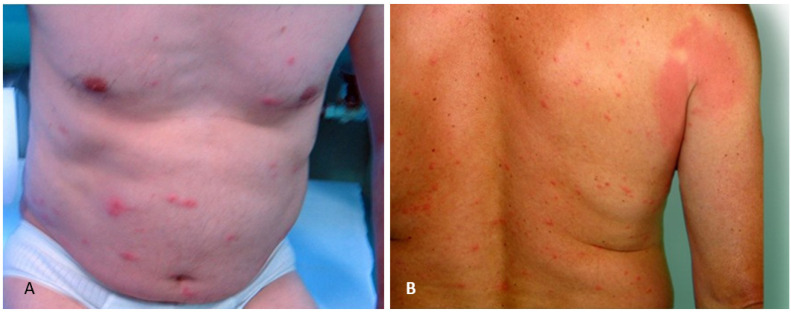
Skin lesions in patients with no co-morbidities bitten by blackflies for the first time. (**A**) Numerous papules and vesicles with serous discharge and erythema on the abdomen and chest in a male patient (case 3); (**B**) Numerous vesicles at the site of blackfly bites and extensive erythema on the arm in a male patient (case 4).

**Case 3**. Male aged 49 years (Figure 4A) with no comorbidities. He was first attacked by blackflies in the afternoon on 3 July 2004 while working in the forest.

The patient had papules and blisters with serous secretions, erythema on the abdomen, single lesions on the chest, and itching, burning, and warming sensation on the extremity. The skin lesions persisted for 7 days.

Treatment: Hydro-cortisone 0.1 i.v., Hydro-cortisone 0.1% cream 2 × a day, antihistamine drug (Clemastin 3 × a day), Calcium C one tablet 3 × a day

**Case 4**. Male aged 57 years (Figure 4B). He was first attacked by many blackflies in the afternoon on 23 June 2004 while taking a rest in the forest. The patient had extensive erythematous skin lesions on the arm and on the back near the axilla, which persisted for 7 days despite the treatment.

Treatment: Hydro-cortisone 0.2 i.v., an antihistamine drug (Loratadine—Claritine one tablet 1 × a day), Calcium 1 tablet 3 × a day.

**Figure 5:**
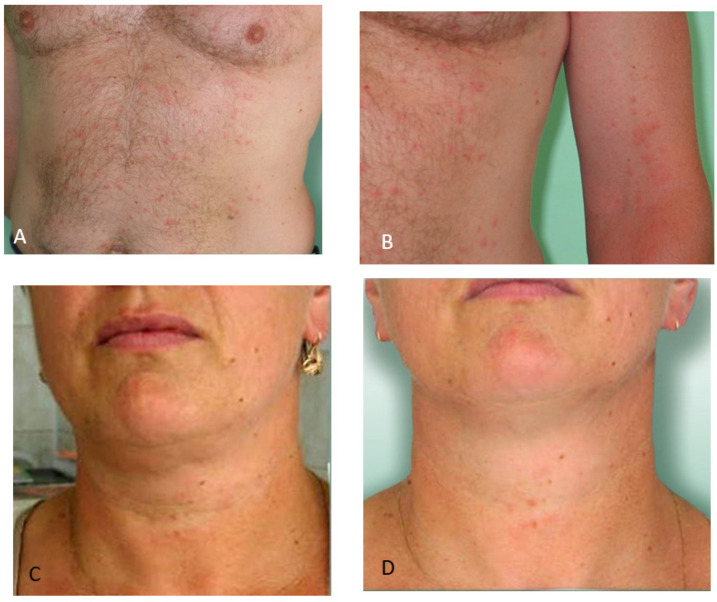
Skin lesions in patients without co-morbidities, attacked by blackflies repeatedly. (**A**,**B**) numerous papules on the abdomen, chest, and upper extremity in a male patient attacked by blackflies repeatedly (case 5); (**C**,**D**) Inflammatory reactions at the site of repeated blackfly bites in the lip area and papules on the neck in a female patient (case 6).

**Case 5**. Male aged 49 years (Figure 5A,B). He had been repeatedly bitten by blackflies during cleaning work in his household at noon on 7 July 2004. The patient had numerous papules on the abdomen, chest, and on the upper limb in the axilla area, with an erythematous lesion in the armpit area persisting for 5 days.

Treatment: Hydro-cortisone 0.1 i.v., Hydro-cortisone 0.1% cream topically, Clemastin 1 tablet 3 × a day, and Calcium C 1 tablet 3 × a day

**Case 6**. Female aged 47 years (Figure 5C,D) attacked by blackflies in the afternoon on 27 July 2004 while working in the garden. Previously, she had been repeatedly bitten by blackflies. The patient had itchy papules on the neck and numerous blackfly bites in the lip area. The skin lesions were visible for 10 days.

Treatment: topical Hydro-cortisone 0.1% cream 2 × a day, Loratadine—Aleric 1 tablet 1 × a day, Calcium 1 tablet 3 × a day.

**Figure 6:**
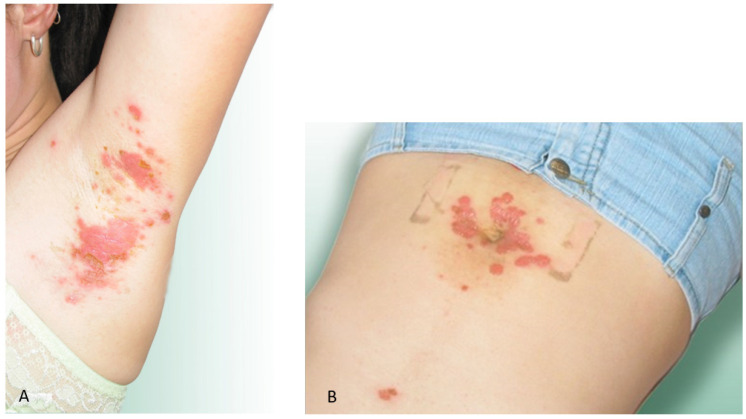
Numerous papules and vesicles. (**A**) in the axillary region and (**B**) on the abdomen, both with secondary bacterial infection in a female patient attacked by blackflies for the first time (case 7).

**Case 7**. Female aged 17 years (Figure 6A,B). She was first attacked by blackflies in the afternoon on 6 July 2004 while working in the garden. The patient presented with numerous papules and vesicles filled with serous fluid on the abdomen, and in the axilla with secondary bacterial infection. The skin lesions persisted for 21 days after the blackfly bites.

Treatment: Elocom 0.1% ointment 1 × a day, an antihistamine drug (Clemastin, 1 tablet 2 × a day) for 20 days, 1 tablet of Calcium 3 × a day, and Neomycin 3% spray topically 3 × a day.

### 3.2. Threat to Human Health Posed by Blackfly Attacks

The respondents varied in age, but the majority were professionally active people aged 36 to 55 (Table 1). The mean age of the females and males was 46.04 ± 18.56 years and 39.72 ± 20.04 years, respectively. Most respondents had primary and secondary education, and much fewer declared higher education. The study group also comprised children and subjects that had not graduated from primary school. The majority of the respondents were manual workers employed in agriculture, horticulture, and forestry. A smaller group consisted of white-collar workers, schoolchildren, students, farmers, retirees, and pensioners.

As indicated by 84.2% of the respondents, the blackfly attacks occurred in areas with large forest complexes, mainly in coniferous and mixed forests. Approximately 52.2% of the respondents were attacked by blackflies in areas without large water reservoirs but with numerous watercourses (56.2%).

In southeastern Poland, blackfly attacks were recorded from April to September. Throughout the three-year survey period, the highest number of blackfly bites was recorded from May to July with a peak in June (38.73%) (Table 2). The number of attacks varied, depending on the time of day. The greatest number was recorded between 15:00 h and 18:00 h (37.10%) and between 18:00 h and 22:00 h (33.03%); additionally, incidents of blackfly attacks were recorded at different time points, mainly in the morning (Table 2). The patients were attacked outdoors in both rural and urban areas. However, the most frequent blackfly attacks occurred in fields (31.07%), coniferous and mixed forests (22.14%), and households (19.11%) (Table 1). Over half of the survey respondents (54.5%) reported incidents of blackfly bites occurring while they were involved in occupational activities (work in the field, in the forest, and on their farms), while the other participants (45.5%) were attacked during recreation (passive rest in the blackfly habitats, sport activities, or playing as in the case of children) (Table 1).

We did not find a correlation between the persistence of the skin lesions and the age of the patient. In the different age groups, the lesions resolved within 3.63 ± 1.58 days to 3.94 ± 4.15 days after blackfly bites.

**Figure 7:**
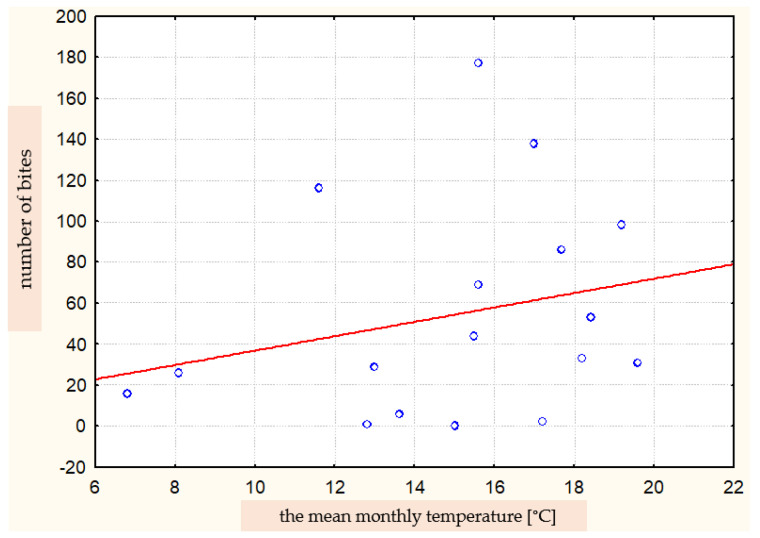
Correlation between the number of bites in a given month and the mean monthly temperature (Spearman’s rank correlation coefficient R = 0.359).

There was no significant correlation between the number of bites and the mean monthly temperature (Spearman’s correlation coefficient R = 0.359) (Figure 7). The data may indicate a greater complexity of this phenomenon. In addition to the mean monthly temperature, the number of bites was probably also influenced by other meteorological factors.

**Figure 8:**
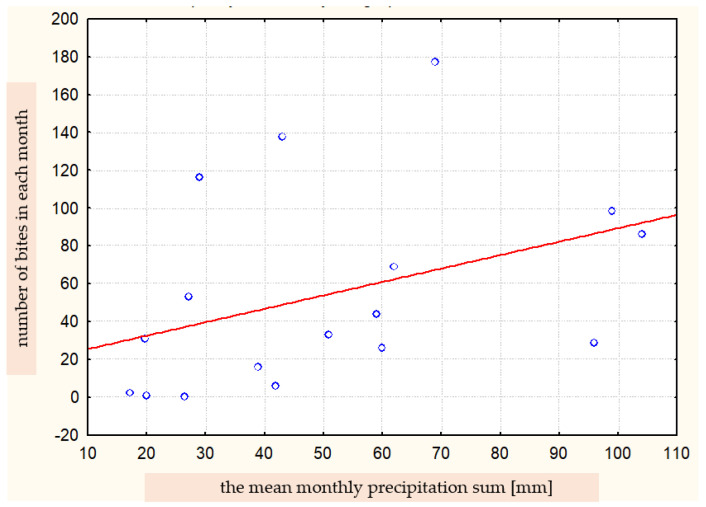
Correlation between the number of bites in a given month and the mean monthly precipitation sum (Spearman’s rank correlation coefficient R = 0.550).

The statistical analysis showed a correlation between the number of bites and the mean monthly precipitation sum at the significance level of *p* = 0.022 (Figure 8). The value of Spearman’s correlation coefficient was R = 0.550.

**Figure 9:**
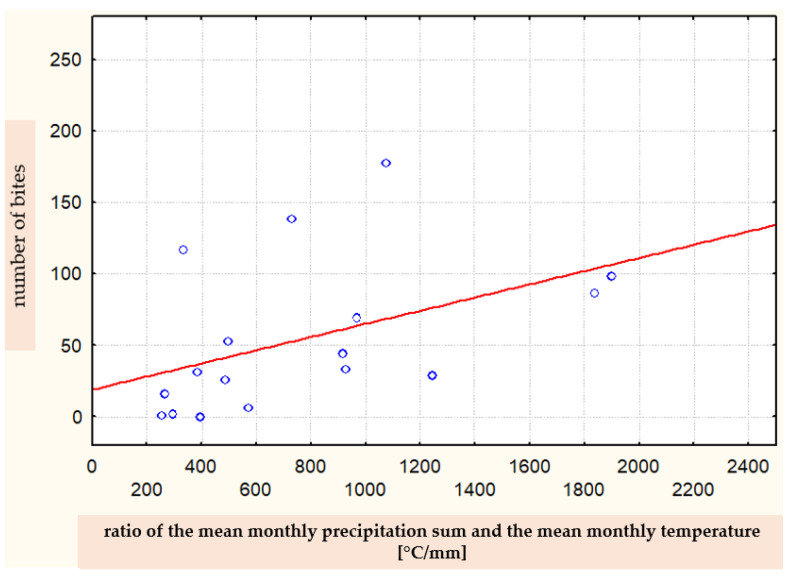
Correlation between the number of bites in a given month and the ratio of the mean monthly temperature and the mean monthly precipitation sum (Spearman’s rank correlation coefficient R = 0.590).

In contrast, the statistical analysis showed a significant (*p* = 0.013) relationship between the number of bites and the ratio of the mean monthly temperature and the mean monthly precipitation sum (Spearman’s correlation coefficient R = 0.590) (Figure 9).

## 4. Discussion

Among the 2330 species of blackflies identified in the world, 230 species occur in Europe [2], with 49 species in Poland [33]. Eight species of blackflies in the Polish fauna occur massively and attack humans and animals. *Simulium (Boophthora) erytrocephalum*, *Simulium (Schoenbaueria) pusillum*, *Simulium (Simulium) reptans*, *Simulium (Simulium) ornatum*, and *Simulium (Wilhelmia) equinum* are regarded as the most troublesome species [34].

Within ca. 4–5 min, female blackflies ingest from approx. 1.08 to 3.26 µL of host’s blood [3], simultaneously injecting many pharmacologically and immunologically active molecules [35,36,37,38,39,40,41,42,43,44,45,46]. The severity of skin lesions caused by parasitic blackflies is determined not only by the components of their saliva, but also by the feeding mode. Female blackflies tear host skin and blood vessels with their mouth organs, thus contributing to an increase in the size of the lesion and spread of bioactive components of saliva in the attachment site.

The mode of feeding of female blackflies consists in rupture of host’s skin and blood vessels with their mouthparts and injection of the toxic components of saliva during blood ingestion, which activate various pathological mechanisms.

Although many incidents of blackfly bites on humans and skin lesions caused by these insects have been reported worldwide [47,48,49,50,51,52,53,54,55,56,57], such cases have been monitored [58,59,60] and epidemiological studies have been conducted [61,62] in a few regions. In Poland, insufficient attention has been paid so far to the effects of the parasitism of blackflies on humans [32,63] despite the wide spread and mass occurrence of these insects in some regions [33,34,63,64,65].

Besides blackflies, residents of eastern Poland are attacked by other parasitic arthropods, most frequently by the castor bean tick *Ixodes ricinus* (Ixodida: Ixodidae) or mosquitoes (Diptera: Culicidae) and much less often by the deer ked *Lipoptena cervi* (Diptera: Hippoboscidae) [66], whose presence was not confirmed in the areas indicated by the patients and inspected during the field studies. In eastern Poland, blackflies are the only arthropods severely attacking people and leaving skin wounds with edges torn by their mouth organs. The feeding sites of *I. ricinus* and *L. cervi* are characterized by a clearly visible puncture with erythematous infiltrated non-demarcated lesions, usually resolving after a few days, and an invisible or poorly visible puncture with irregularly shaped scattered erythematous papules persisting from several weeks to a year, respectively [66]. In turn, mosquitoes most often do not produce a lesion at the bite site; less often, there may be a poorly visible puncture surrounded by reddening, which usually disappears within one to two hours (our own observations).

As shown by our survey carried out in the southeastern part of Poland, blackfly bites induce solely skin lesions in as many as 88.52% of patients and both skin and systemic symptoms in only 11.48% [32]. The blackfly bite symptoms described previously were observed in patients with a good health status with no comorbidities.

Many case reports described in the literature from the beginning of the 20th century (e.g., [67,68,69]) to the beginning of the 21st century [32,56,57,70] show highly diverse reactions in humans bitten by blackflies and, consequently, a wide spectrum of local and systemic symptoms. In addition to the individual traits and sensitivity of patients, the diversity of symptoms may also be associated with the biological and physiological features of these insects, probably the composition of the saliva of various species. During feeding, female blackflies rupture host’s skin and blood vessels with their mouth parts and inject toxic components of saliva during blood ingestion, which activates various pathological mechanisms.

Most often, lesions such as grouped small pruritic papules, vesicles, erythematous wheals with various ranges, and small crusted ulcerations resulting from a hypersensitivity reaction to simuliid bites are observed [32,46,53,70,71,72]. The dominant systemic symptoms include headaches, elevated body temperature, and lymphadenopathy [32,56,67,70]. Additionally, symptoms of anaphylaxis [71,73] and acute cardiotoxicity [73] may appear. Farkas [62] distinguished variable clinical forms of simuliosis in humans, e.g., edematous, erythematous-edematous, erysipeloid, inflammatory-indurative, phlegmonoid, and hemorrhagic forms.

The signs and symptoms may develop in patients in response to the saliva components at different time points after the blackfly bite. For instance, pruritic dermatitis with considerable edema was detected in a 25-year-old man from Iran as early as within a few minutes after *Simulium kiritshenkoi* bites, while other manifestations, e.g., swollen lymph nodes, joints aching, and 40 °C fever, were noted after a few hours [56]. In turn, an erythematous macule with itching and edema developed the next day after *Simulium quinquestriatum* bite in a 49-year-old male from Japan. The strong inflammatory reactions in this patient persisted for 2–3 days [55].

The skin lesions in the patients described in this study differ from other cases observed in our previous studies in intensity of symptoms [32]. The strong and long-lasting reaction to the components of the blackfly saliva, i.e., numerous itchy papules and large intense erythema, was probably influenced by the patient’s health status, especially long-term cardiovascular diseases (case 1). Special importance may be ascribed to the presence of chronic venous insufficiency, which is one of the most common circulatory system diseases in Western countries occurring in <1–40% in females and <1–17% in males, depending on the geographic location [74]. In Poland, the prevalence of this disease in females may be as high as 84% [75]. In the case described in the present study, the blackfly saliva components with a broad spectrum of activity probably intensified the symptoms of varicose disease. Wach [65] observed erosions and ulcerations of the shin in blackfly-bitten patients with impaired venous circulation.

Pathological dilation of superficial veins in the lower extremities promotes rapid and effective ingestion of blood from vessels damaged by the blackfly mouth organs and enzymes contained in blackfly saliva, e.g., apyrases degrading adenosine diphosphate (ADP) and adenosine triphosphate (ATP) (agonists of platelet and neutrophil aggregation), hyaluronidases catalyzing the degradation of hyaluronic acid and thereby facilitating the penetration of other active components of saliva at the feeding site, proteases increasing the proteolysis rate, vasodilators, platelet aggregation antagonists involved in maintenance of homeostasis, and inflammation agonists [40,46,76,77].

It seems that the development of blackfly bite lesions in the patients with comorbidities was enhanced by factors predisposing to immunity suppression. These include metabolic disorders manifested by obesity, old age, and strong stressors to which they were exposed in their environment. The severe symptoms described in this study in the 65-year-old female patient (case 1) developed after multiple blackfly attacks and persisted for up to 4 weeks despite the treatment. In another patient (case 2), who had a shorter history of cardiovascular disease and was attacked by blackflies once, the skin lesions resolved after only 3 days.

The differences in the symptoms and course of simuliosis in the patients presented in this study and in other cases described in the literature are probably associated with individual traits and sensitivity and with the biological and physiological characteristics of blackflies, e.g., the composition of saliva of different species.

The shin and forearm were the most frequently attacked body regions in patients with severe symptoms, whereas the frontal and cheek areas on the head were attacked rarely. Skin lesions caused by blackfly bites are most often located on uncovered upper and lower extremities, as observed in clinical practice, and this location has been most frequently described in the case reports cited in the present study [32,52,53,56,61,62,65,70,72]. Blackfly bites on lower extremities (mainly on shins) accounted for 48.83% and 25.31% of all attacks of these insects in females and males in southeastern Poland, respectively [32].

In most of the reported cases, skin lesions induced by blackfly bites resolved after a few days (e.g., [62,65,72]), less often after two weeks, and after 20–25 days when no treatment was administered [52]. In patients with no co-morbidities from southeastern Poland, skin lesions persisted for an average of 3.84 ± 2.99 days in females and 3.68 ± 2.21 days in males [32].

The severe reactions to the blackfly bites persisted from 3 days to 28 days. No relationship was found between patients’ age and the duration of the persistence of simuliosis symptoms. In our previous epidemiological and clinical studies, we found no correlations between the picture of skin lesions, systemic symptoms, and the number and frequency of blackfly bites. There was no correlation between the persistence of local skin signs and systemic symptoms and the sex of patients [32].

The skin lesions in the patients described in this study caused great discomfort and impeded patients’ daily activities. Similarly, other authors have reported that inflammatory reactions and erythema in the lower extremities may hinder activity [52,56] and mobility, even in young patients [53].

Mechanical damage to the human skin caused by the mouth parts of female blackflies may promote infection with bacteria present in the environment or on the blackfly body. Chomicz et al. [78] isolated numerous pathogenic bacterial strains from homogenates of blackfly heads and abdomens, with predominance of *Enterococcus faecium* and *E. faecalis* (Gram-positive fecal streptococci) and *Enterobacter cloacae* (Gram-negative intestinal bacteria).

Due to the penetration of bacteria through the skin damaged by the blackfly mouthparts and/or by scratching the itchy bite site, the skin lesions were intensified and the treatment period was extended to 21 days.

The increase in the number of cases of blackfly attacks observed in southeastern Poland may be caused by the expansion of the blackfly occurrence range and the increase in the population size associated with climate warming (on average by 0.6–0.8 °C/year) and warm winters [79]. The development of larvae and pupae releasing adult blackflies is also supported by the improvement of the purity of Polish waters [33]. Hydrometeorological conditions play a key role in the development of blackflies [16,80], as a greater part of their life cycle takes place in water. Blackfly larvae and pupae develop in rivers and streams with fast currents.

The movement of adult blackflies over certain distances is associated with atmospheric conditions—air temperature, sunlight, vapor pressure deficit, and wind strength [33,81,82]. Changes in the occurrence range, dynamics of the activity, and behavior of these insects depending on the climate and weather conditions should therefore be taken into account in strategies for prevention of blackfly bites. Despite the climate changes observed in eastern Poland, the seasonal and daily rhythms of blackfly activity have not changed during the last 18–20 years (unpublished data). Similar rhythms of blackfly activity observed by analysis of reported cases of attacks on humans were also recorded in 2009–2015 in Zaragoza (Spain) [83]. However, as indicated by these researchers, the number of patients requiring medical care after blackfly bites has increased in the analyzed area.

In the eastern region of the EU, there is a high risk of simuliosis from May to July, i.e., a period when people often stay outdoors. Farmers and forest workers who perform seasonal work in blackfly habitats and subjects practicing sports or hiking in the afternoon and evening, i.e., during the peak daily activity of these insects, are most vulnerable to attacks from these insects. The differences in the frequency of blackfly attacks of males and females in the different areas noted in the present study are probably associated with the type of outdoor activities. Women in Lubelskie Province, which is one of the poorest regions in the European Union, are more likely to do field work and pick forest fruits and mushrooms, which are their main source of income.

The habitats of adult blackflies, i.e., mainly forest areas and meadows near rivers and streams, may also be colonized by other blood-sucking arthropods that cause skin inflammation in humans, e.g., deer keds (*Lipoptena cervi*) [84,85,86] and *Ixodes ricinus* ticks [87,88,89]. Therefore, correct identification of skin lesions in patients is essential for diagnostic and therapeutic procedures in clinical practice. Information about potential biological threats to human health in different regions is also useful for development of strategies for protection of the health of residents.

Since female blackflies often attack humans staying outdoors for recreational and occupational purposes, it is advisable to use methods for limitation of their population size, mainly biological methods [90,91,92], in areas of the mass occurrence of these insects, and to popularize protective measures against their bites [93,94,95].

## 5. Conclusions

Factors that may influence the pathogenesis and course of simuliosis in humans have not been clarified to date. However, as suggested in the present study, the course of simuliosis in patients may depend on the co-occurrence of bacterial infections, cardiovascular diseases (mainly chronic venous insufficiency), metabolic disorders, and the effects of long-term stress-inducing stimuli that weaken immunity. Therefore, special focus should be placed on elderly patients and other subjects with chronic diseases in areas of mass occurrence of blackflies. The insufficient knowledge of the causes of the intensification of blackfly bite symptoms in humans suggests that the various clinical cases of simuliosis in patients should further be analyzed.

Besides the patient’s health status, the frequency of exposure to blackfly bites and individual traits may be the determinants of the development of severe reactions to the components of blackfly saliva. This issue, however, requires further investigations.

During peaks of daily and seasonal activity of blackflies, mainly in the afternoon hours in June, staying in areas with abundant occurrence of these insects should be limited and repellents should be used as a means of personal protection. It is also necessary to monitor the occurrence of blackflies in various habitats and to develop a strategy to protect humans from attacks by these insects.

## Figures and Tables

**Table 1:** Socioeconomic background and circumstances of black fly bites in studied patients from Lubelskie Province (southeastern Poland) (2003–2005, *n* = 418).

Viable	Females		Males		Total	
	*n*	(%)	*n*	(%)	*n*	(%)
**Education**	-	-	-	-		
primary	-	-	-	-	175	41.87
secondary	-	-	-	-	173	41.39
higher	-	-	-	-	39	9.33
others	-	-	-	-	31	7.41
**Profession**	-	-	-	-		
laborer	-	-	-	-	136	32.53
brain worker	-	-	-	-	88	21.1
housewife	-	-	-	-	68	16.3
pensioner	-	-	-	-	46	11
student	-	-	-	-	72	17.22
child	-	-	-	-	8	1.91
**Age**						
0–7	5	1.2	7	1.7	12	2.9
8–14	9	2.15	18	4.31	27	6.46
15–25	29	6.94	27	6.46	56	13.4
26–35	26	6.22	15	3.58	41	9.8
36–55	108	25.84	46	11	154	36.84
56–65	40	9.57	34	8.13	74	17.7
66–89	39	9.33	15	3.59	54	12.92
**Circumstances of bite**						
occupational exposure	152	36.53	74	17.79	226	54.32
recreational exposure	117	28.12	73	17.55	190	45.67
**Area of attack**						
field/garden	94	21.91	42	9.79	136	31.7
forest	69	16.08	26	6.06	95	22.14
estate	40	9.32	42	9.79	82	19.11
meadow	32	7.45	20	4.67	52	12.12
orchard	16	3.72	11	2.57	27	6.29
park	1	0.23	32	1.87	9	2.1
playground	1	0.23	11	1.87	12	2.8
others	9	2.09	7	1.64	16	3.73

*n*—number of patients; - no data.

**Table 2:** Seasonal and diurnal occurrence of blackfly bites noted in Lubelskie Province (southeastern Poland (2003–2005, *n* = 418).

Viable	Females		Males		Total Number of Bites	
	*n*	(%)	*n*	(%)	*n*	(%)
Month of bite						
April	33	3.56	11	1.19	44	4.75
May	109	11.76	105	11.32	214	23.08
June	184	19.85	175	18.88	359	38.73
July	109	11.87	106	11.43	215	23.19
August	56	6.04	32	3.45	88	9.49
September	4	0.43	3	0.32	7	0.75
	**Females**		**Males**		**Total number of attacks**	
	***n***	**(%)**	***n***	**(%)**	***n***	**(%)**
Day time of the attack						
early morning	53	11.99	24	5.43	77	17.42
morning	16	3.62	18	4.07	34	7.69
midday	12	2.71	7	1.58	19	4.29
afternoon	97	21.94	67	15.16	164	37.1
evening	89	20.13	57	12.9	146	33.03
night	1	0.23	1	0.23	2	0.45

*n*—number of patients.

## Data Availability

The data presented in this study are available on request from the corresponding author. The data are not publicly available due to patient privacy.
